# Near Infrared Characterization of Hetero-Core Optical Fiber SPR Sensors Coated with Ta_2_O_5_ Film and Their Applications

**DOI:** 10.3390/s120202208

**Published:** 2012-02-15

**Authors:** Keiju Takagi, Kazuhiro Watanabe

**Affiliations:** Department of Information Systems Science, Faculty of Engineering, Soka University, 1-236, Tangi-machi, Hachioji-shi, Tokyo 192-8577, Japan; E-Mail: eng-office@t.soka.ac.jp

**Keywords:** optical fiber sensor, hetero-core structure, surface plasmon resonance, tandem-connected, multi-point measurement

## Abstract

This paper describes the characteristics of optical fiber sensors with surface plasmon resonance (SPR) at 1,310 nm in which the scattering loss of silica optical fiber is low. SPR operation in the infrared wavelength range is achieved by coating a thin tantalum pentaoxide (Ta_2_O_5_) film. The novelty of this paper lies in the verification of how the hetero-core scheme could be operated as a commercial base candidate in the sense of easy fabrication, sufficient mechanical strength, and significant sensitivity as a liquid detector under the basis of a low loss transmission network in the near infrared wavelength region. The effect of Ta_2_O_5_ layer thickness has been experimentally revealed in the wavelength region extending to 1,800 nm by using the hetero-core structured optical fiber. SPR characterizations have been made in the wavelength region 1,000–1,300 nm, showing the feasible operation at the near infrared wavelength and the possible practical applications. In addition, the technique developed in this work has been interestingly applied to a multi-point water-detection and a water-level gauge in which tandem-connected SPR sensors system using hetero-core structured fibers were incorporated. The detailed performance characteristics are also shown on these applications.

## 1. Introduction

In the fields of medicine and biology, optical sensors based on surface plasmon resonance (SPR) have been developed as high-resolution refractive index sensors which are sensitive to small changes in refractive index induced due to events such as the combination of antigens and antibodies. Many of these sensors have utilized a prism type coupling based on the so-called Kretschmann configuration [1–4]. Optical waveguides [5,6] and grating couplers [7,8] were also proposed as alternatives to the prism systems for SPR. Recently, many optical fiber SPR sensors have been reported [9–11] since optical fibers offer us one of the simplest optical waveguides which could be useful tools to achieve remote sensing and sensor miniaturization demanded in the applications operating in outdoor conditions. The history of SPR optical fiber sensors tells us that elaborative works had previously to be made to establish how to access to environmental conditions outside core with utilizing evanescent waves that is built up due to total reflection at the core/cladding boundary region. Therefore, in order for the optical signal inside the core to interact directly through evanescent waves arising from the core/cladding boundary region, the fabrication of core-exposed structures, so-called uncladding (or tapered) fiber, has been attempted by eliminating thick cladding layers. Removing cladding layers is a highly complex task, and the resulting structures have less mechanical strength which makes them unsuitable for practical usage.

On the other hand, it is known that most of SPR sensors could be operated in the visible range in an aqueous environment with a refractive index close to 1.33. In addition, optical fiber sensors based on SPR could also be operated in the limited wavelength in the range 600–700 nm under a similar environment [9–11] as well. This is because that SPR excitation condition is derived from the relationship between the thickness of thin metal film, the angle of incident light and the refractive index of silica. In contrast to those visible range operation, it was demonstrated by Homola *et al*. that the resonance wavelength of SPR excitation could be shifted toward longer wavelengths by coating a high-refractive index thin film such as tantalum pentaoxide (Ta_2_O_5_) [12,13] in addition to the conventional SPR metal coating. They demonstrated a SPR sensor operating at 825 nm for refractive index measurements of aqueous environments employing a Ta_2_O_5_ coated single-mode optical fiber whose cladding layer was removed [14,15]. Their results showed that the thicker the Ta_2_O_5_ coating the longer the operating wavelength becomes. An advantage would accordingly be obtained in a SPR fiber optic sensor working at the near infrared wavelengths over 825 nm extending to 1310 nm, where silica fibers shows lower transmission loss. In particular, the use of 1310 nm allows a flexible system arrangement using a versatile selection of LED light source and PD detectors.

The authors have previously reported a variety of hetero-core structured fiber optic SPR sensors [16–18]. Favorable characteristics of the hetero-core SPR sensor include its simple structure with easy fabrication which has no need of cladding removal and its simple interrogation method based on the intensity mode operation owing to a broader SPR spectrum of multi-mode transmission fibers (MMF). Another advantage of this SPR sensor can be found in the flexibility in the change of the insertion loss, therefore the sensitivity by adjusting the length of sensor portion. The novelty of this paper is to verify how the hetero-core scheme could be operated as a commercial base candidate in the forms of easy fabrication, sufficient mechanical strength, and significant sensitivity as a liquid detector on the basis of a low loss transmission network in the near infrared wavelength region.

In this paper, the effect of Ta_2_O_5_ layer thickness has been experimentally revealed in the longer wavelength region extending to 1,800 nm by using the hetero-core structured optical fiber. SPR characterizations have been made in the wavelength region 1,000–1,300 nm, demonstrating feasible operation at the near infrared wavelength and the possible practical applications. In addition, the technique developed in this work has been interestingly applied to a multi-point water-detection system and a water-level gauge in which a tandem-connected SPR sensor system using hetero-core structured fibers was incorporated. The detailed performance characteristics are also shown in these applications.

## 2. Tuning an Operation Wavelength of a Hetero-Core Optical Fiber Based on SPR Using Tantalum Pentaoxide Film

### 2.1. Hetero-Core Structured Optical Fiber SPR Sensor

**Figure 1.**
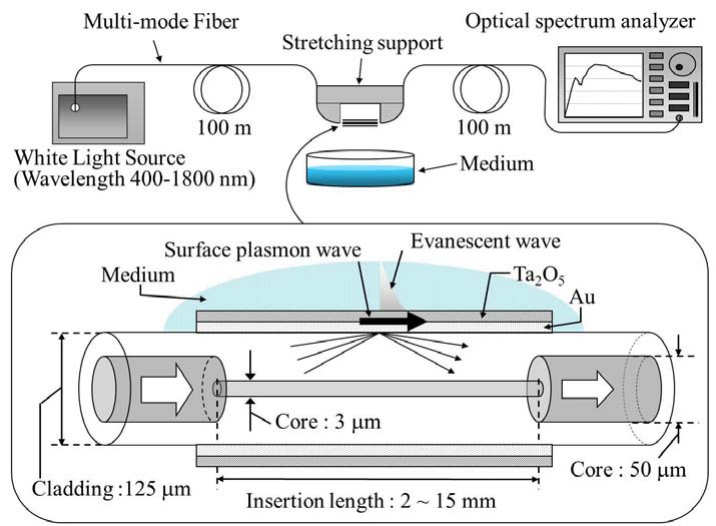
Hetero-core structured optical fiber SPR sensor and the experimental setup to measure the transmitted light spectra.

A hetero-core structured optical fiber SPR sensor is shown schematically in Figure 1. The hetero-core optical fiber consisted of a transmission line multimode (GI) fiber and an inserted segment of a single-mode (SI) fiber, which works as a sensor region. Details of hetero-core SPR sensors were described in our previous works [16,17]. With this structure, since the core diameter of the sensing part was much smaller than that of transmission line fiber, the propagated light wave in the transmission line would largely leak into the cladding layer of the sensor region. When the leaked light is reflected at the boundary of the cladding layer and the surrounding environment under the condition of internal total reflection, such a light wave induces an optical evanescent wave at the surface of the cladding layer, hence SPR excitation by coating the cladding surface with a thin metal film, as shown in Figure 1, similar to the case of the conventional Kretschmann configuration sensor.

A light wave traveling through the sensor region could partially re-couple into the core of latter transmission line. Additionally, since light in the transmission line propagates in multimode forming numerous modes, many SPR spectra are induced corresponding to various incident angles. As a result, an output spectrum could be overlapped to be a broader spectrum due to many excited resonance wavelengths. This broader spectrum makes possible flexible usage in which the intensity mode of operation is easily made with a fixed wavelength of probing. No coating on the hetero-core produces only a transparent spectrum curve without any SPR resonance spectrum.

### 2.2. Experimental Set-Up to Estimate Ta_2_O_5_ Thickness Dependence

A sensor for the experiment was fabricated with a GI MMF with core and cladding diameters of 50 and 125 μm, respectively, and an inserted sensitive region of a 15-mm-long SI SMF with a core diameter of 3 μm and the same cladding diameter of 125 μm as the GI fiber. The fabricated hetero-core region was then cylindrically Ta_2_O_5_-coated with thicknesses of 0–60 nm over a 25-nm thick Au first layer by using an RF sputtering machine (CFS-4DS-231, Shibaura Mechatronics Corp.), in which a specially designed rotating mechanism was devised to symmetrically realize uniform deposition on the cladding surface. According to our previous work [17] for the Au-SPR sensor, the Au thickness around 20 nm offered us the larger SPR dip, but the smaller SPR shift due to the change of refractive index. The Au/Ta_2_O_5_ coating combination was properly selected with 25-nm Au to give a sufficient SPR dip according to a series of experiments. The experimental set-up of Ta_2_O_5_ thickness characteristics consisted of a halogen lamp, whose wavelength was in the range of 400–1,800 nm, and an optical spectrum analyzer (AQ6315A, Ando Electric Co., Ltd.), as shown in Figure 1. The output from the halogen light source was guided into a 100-m upstream transmission fiber and finally introduced to the spectrum analyzer after traveling in a downstream 100-m fiber through the sensing part. Considering the possible usage of this sensor in the outdoors full-scale experiment where several tens of fiber extension could be required, 100-m fibers are connected before and after the sensor element. The characteristics obtained in this work are reasonably reserved regardless the length of in- and out-transmission fiber as long as multi-mode is fully excited in a stable state, except that the offset level of transmission loss could be changed with the length. In order to avoid unfavorable fluctuation due to mode disturbance of MMF, the sensor region was firmly mounted in a straight line configuration with a stretching support. In the series of experiments, glycerin solutions were prepared as test liquids with concentrations of 0–50% by weight, corresponding to refractive indices ranged 1.333–1.398 RIU.

### 2.3. SPR Effects by Coating Ta_2_O_5_

**Figure 2.**
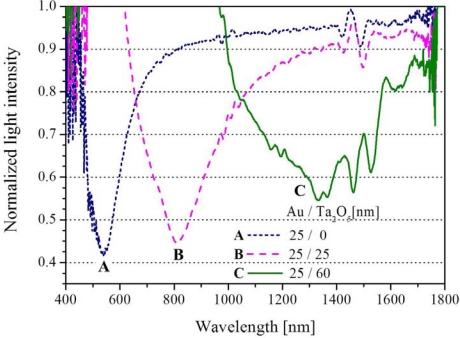
Experimental SPR spectra in water normalized with air at various film thickness of Ta_2_O_5_ (A: Without Ta_2_O_5_, B: 25 nm, C: 60 nm) deposited on gold film (25 nm).

**Figure 3.**
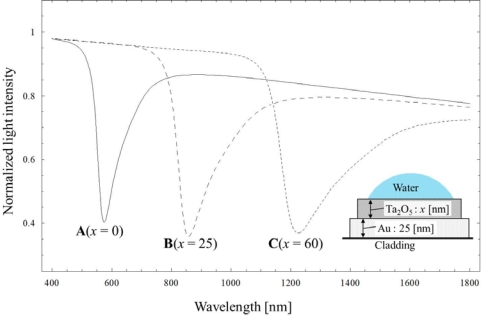
Theoretical SPR spectra in water at various film thicknesses of Ta_2_O_5_ (A: Without Ta_2_O_5_, B: 25 nm, C: 60 nm) on gold film (25 nm).

Figure 2 shows SPR spectra A, B and C experimentally obtained in the case of water for the sensors having Ta_2_O_5_ thicknesses of 0, 25 and 60 nm, respectively. These results are normalized with the spectrum for air. As the result of increasing the Ta_2_O_5_ thicknesses extending to 60 nm, the experiment shows that the resonance wavelength is largely sifted to the near-infrared wavelength region over 1,000 nm. Although each spectrum is somewhat uneven at the wavelength around 1,400–1,600 nm, this could be caused by an interference effect between longer wavelength modes when the light wave re-couples into the core downstream, since the same results can be appeared in the case of no coating hetero-core without water. Figure 3 shows theoretical SPR spectra based on multi-layered model [19,20] for three Ta_2_O_5_ thicknesses, assuming that there would be a multi-mode distribution [17,18] with the corresponding incident angles in the hetero-core structure. The refractive indices of each layer at 1,310 nm are given as Au: 0.413 RIU, Ta_2_O_5_: 1.888 RIU using the wavelength dependence previously reported [20,21].

**Figure 4.**
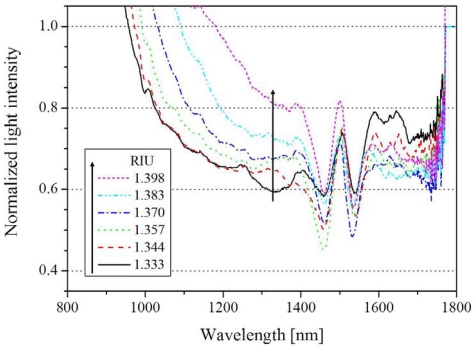
Experimental spectra in near infrared wavelength region at various refractive index of the solution. (The film thickness of gold and Ta_2_O_5_ were 25 nm and 60 nm, respectively).

Comparing the experimental result (Figure 2) with the numerical calculation (Figure 3), it is indicated that the wavelength-shifts are found to be similar for the increases of Ta_2_O_5_ thickness. In this work, the coating of 25/60 (Au/Ta_2_O_5_) shows sufficient resonant absorption at the wavelength around 1,310 nm, although slight differences are seen between the experimental and the theoretical results. The Au/Ta_2_O_5_ coating combination was properly selected to give a sufficient SPR dip according to a series of experiments. The thicker the Ta_2_O_5_ coating is, the broader and more shallow the SPR spectrum becomes. This work used only Ta_2_O_5_ coating as one of high refractive index materials since the purpose of this scheme is firstly to show the capability as a liquid detector rather than to attempt possible other materials previously reported [22]. Figure 4 shows the SPR spectrum change with various refractive indices of liquids for the 25/60 coating, under the condition that the resonant wavelength appeared around 1,300 nm because of the use of 60 nm-Ta_2_O_5_ coating.

These results were normalized with the spectrum for air. The resonant absorption in the wavelength range of 1,000–1,400 nm decreases with the increase of refractive index, showing that the same interference effect as in Figure 2 appears in the 1,400–1,600 nm range. As can be seen from Figure 4, probably because of such interference effect, the intensity change in the wavelength region longer than 1,300 nm is heavily disturbed in a complicated manner, hence with an ambiguous resonant shift. The sensitivity can be derived as 1.3 dB per 0.065 RIU at 1,310 nm. The sensitivity obtained is comparable to that in the previous works [10,11,17,22]. The data accordingly shows that the wavelength-shifted hetero-core scheme can be used more favorably as liquid detection, rather than as a refractive index (RI) measurement. The obtained experimental results indicate that an attractive liquid detection sensor could be realized for liquids with refractive indexes of 1.333–1.398 RIU such as water or ethanol.

## 3. Water Detection Using the Developed SPR Sensors

### 3.1. Multi-Point Water Detection

**Figure 5.**
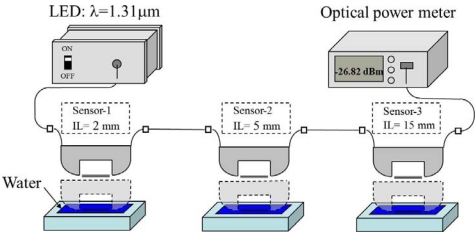
Experimental setup for multi-point liquid detection system.

The absorption loss of the hetero-core SPR sensor can be reproducibly adjusted by changing the insertion length [23]. Since SPR interaction occurs effectively with longer insertion lengths, the sensitivity of the sensor is increased. However, the insertion loss is also increased. The insertion length thus shows a trade-off between sensitivity and insertion loss. Taking advantage of the flexible change of SPR loss, authors have developed a multi-point liquid detection system using three different sensors tandem connected in a single line, in which the three sensors can be identified. In order to separately interrogate the state of each sensor, specified losses were given to each sensor by tuning the insertion length. Sensor detection succeeded only with a combination of a light source of LED and a photo diode. Since this multi-point detection system uses no OTDR, hence no data averaging which is a time consuming process, the detection can be made on a real-time base. The experimental setup for multi-point liquid detection was arranged as shown in Figure 5, and consisted of three hetero-core SPR sensors connected in tandem, an LED (−19.5 dBm) whose wavelength is 1,310 nm as a light source and an optical power meter (PD: OP710, Opto Test Co.).

**Figure 6.**
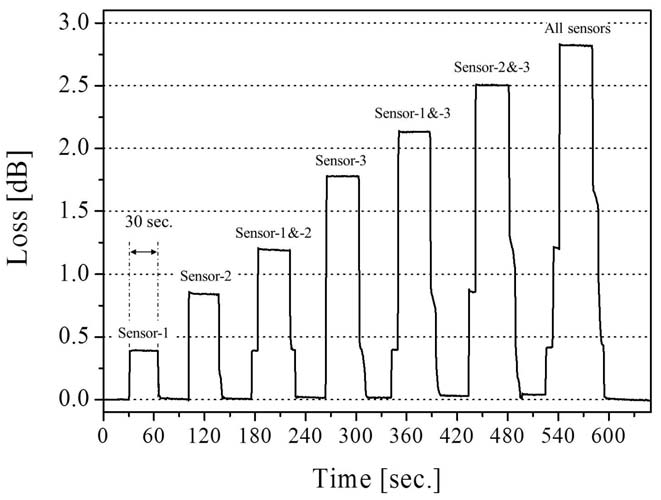
Real-time response in optical loss for each detection pattern.

**Figure 7.**
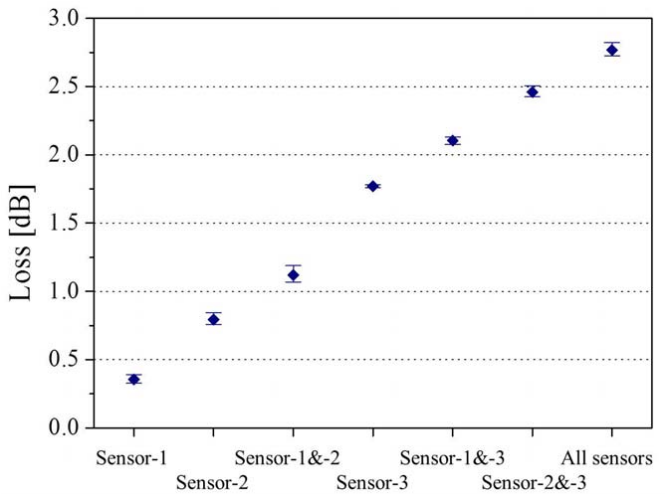
The average of optical loss of each pattern with these five trials.

The output from the LED was guided into a transmission fiber and finally introduced to the PD through the three sensors. The insertion lengths (IL) of the three sensors, denoted as sensor 1, 2 and 3, are 2, 5, and 15 mm, respectively. In the experiments, seven detection patterns were tested as shown in Figure 6. Depending on the sensor combination, extinguished losses are indicated. The losses for each combination were measured as the optical intensity for 30-second, then all sensors were exposed to air for 30-second before the next test. In this way, the losses for all patterns were continuously changed in the order of loss increase with the sampling frequency of 1.67 Hz. Figure 6 shows the real-time response of optical loss change in all patterns. As can be seen, sensor combination is found to be shown as the different levels of loss. In order to evaluate the repeatability of the system, the average of optical loss of each pattern over five trials is shown in Figure 7, which shows that the loss detection is reproducible enough to find the sensor combination. As explained above, the feasibility of a multi-point liquid detection system based on LED-PD has been demonstrated by employing the hetero-core optical fiber SPR sensor coated with Au/Ta_2_O_5_ thin metal. In addition, improvement of this multi-point detection system would be expected by giving the loss as the n^th^ power-of-two (a × 2^(n−1)^) interval to an n^th^ sensors.

### 3.2. Water Level Detection

**Figure 8.**
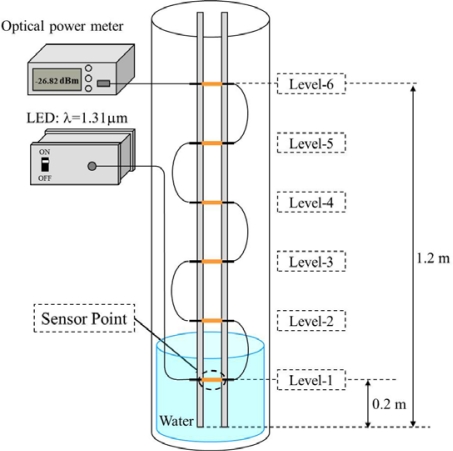
Experimental setup for water-level gauge.

A water-level gauge can be also devised by employing a number of vertically arranged sensors which are connected in tandem, as shown in Figure 8. An identical loss is given to all sensors so that the sum of losses indicates the water level increase. Resolution of the water-level can be given as 0.2 m for this case, and be selected by changing the number of sensors per unit length which are placed in a stepwise form. The measurement system consists of six hetero-core SPR sensors connected in tandem and the same LED-PD combination as seen in Figure 5.

**Figure 9.**
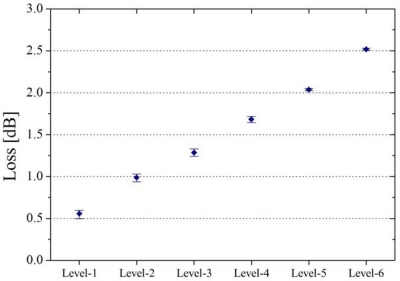
The average of optical loss of each water-level with these five trials.

Figure 9 shows the average of optical loss as a function of the number of sensing points immersed in water, in which these losses were obtained by five consecutive experiments.

Optical losses of each water-level were measured after each sensing head was completely immersed. As can be seen, it is found that the optical loss monotonically increases with the water-level raise. The minimum loss step per sensing head immersed in water is 0.22 dB. The error bars shown in Figure 9 correspond to a reproducibility less than 0.06 m.

## 4. Conclusions

SPR characteristics at the near infrared wavelength region around 1,310 nm have been experimentally demonstrated by means of the hetero-core structured optical fiber technique. Experimental results revealed that the tested fiber sensor is capable of detecting water using an LED/PD system operated at 1,310 nm by coating a 60-nm thick Ta_2_O_5_ layer.

Taking advantage of the results obtained in this work, multi-point water-detection and a water-level gauge have been devised and the corresponding performance characteristics demonstrated. It was shown that the multi-point water-detection application can identify the position of the sensor immersed in water. On the other hand, it was also shown that the water-level gauge can measure a six-staged water-level. The application shown in this work allows us to build an easy system arrangement using a simple LED/PD setting, the obtained Ta_2_O_5_ thickness dependences as the sensor characteristics would therefore provide useful applications from a practical point of view.
